# The comparison of outcomes from tyrosine kinase inhibitor monotherapy in second- or third-line for advanced non-small-cell lung cancer patients with wild-type or unknown EGFR status

**DOI:** 10.18632/oncotarget.8130

**Published:** 2016-03-16

**Authors:** Giuseppe Bronte, Tindara Franchina, Massimiliano Alù, Giovanni Sortino, Claudia Celesia, Francesco Passiglia, Giuseppina Savio, Agata Laudani, Alessandro Russo, Antonio Picone, Sergio Rizzo, Michele De Tursi, Elisabetta Gambale, Viviana Bazan, Clara Natoli, Livio Blasi, Vincenzo Adamo, Antonio Russo

**Affiliations:** ^1^ Department of Surgical, Oncological and Oral Sciences, University of Palermo, Palermo, Italy; ^2^ Medical Oncology Unit-AOOR Papardo-Piemonte, Messina and Department of Human Pathology, University of Messina, Messina, Italy; ^3^ Medical Oncology Unit, A.R.N.A.S. Civico, Palermo, Italy; ^4^ Department of Medical, Oral and Biotechnological Sciences, University “G. D'Annunzio”, Chieti, Italy

**Keywords:** non-small-cell lung cancer, EGFR, tyrosine kinase inhibitor, chemotherapy

## Abstract

**Background:**

Second-line treatment for advanced non-small-cell lung cancer (NSCLC) patients includes monotherapy with a third-generation cytotoxic drug (CT) or a tyrosine kinase inhibitor (TKI). These options are the actual standard for EGFR wild-type (WT) status, as patients with EGFR mutations achieve greater benefit by the use of TKI in first-line treatment. Some clinical trials and meta-analyses investigated the comparison between CT and TKI in second-line, but data are conflicting.

**Methods:**

We designed a retrospective trial to gather information about TKI sensitivity in comparison with CT. We selected from clinical records patients treated with at least 1 line of CT and at least 1 line of TKI. We collected data about age, sex, performance status, comorbidity, smoking status, histotype, metastatic sites, EGFR status, treatment schedule, better response and time-to-progression (TTP) for each line of treatment and overall survival (OS).

**Results:**

93 patients met selection criteria. Mean age 66,7 (range: 46–84). M/F ratio is 3:1. 39 EGFR-WT and 54 EGFR-UK. All patients received erlotinib or gefitinib as second-line treatment or erlotinib as third-line treatment. No TTP differences were observed for both second-line (HR:0,91; *p* = 0,6333) and third-line (HR:1.1; *p* = 0,6951) treatment (TKI vs CT). A trend of a benefit in OS in favor of 3rd-line TKI (HR:0,68; *p* = 0,11).

**Conclusions:**

This study explores the role of TKIs in EGFR non-mutated NSCLC patients. OS analysis highlights a trend to a benefit in patients who received TKI in third-line, even if this result is statistically non-significant. Further analysis are needed to find an explanation for this observation.

## INTRODUCTION

In the last few years we have witnessed a rapid evolution and expansion of systemic anti-cancer treatments available for patients with advanced non-small-cell lung cancer (NSCLC), whose prognosis has been very poor, with 1−, 2−, and 5-years survival rates of 19%, 8%, and 4%, respectively. Targeted therapy became the new standard of care in a subgroup of patients selected according to their tumor molecular profile. Both epidermal growth factor receptor (EGFR) and EML4-ALK tyrosine kinase inhibitors (TKI) are currently recommended as the best treatment option for patients whose tumors harbor EGFR sensitizing mutations or EML4-ALK translocations, respectively, resulting in a significant improvement of survival outcomes, which nearly doubled compared to standard chemotherapy [1–4].

For the majority of NSCLC patients without a targetable oncogene driver, platinum-based combinations, including gemcitabine and pemetrexed/bevacizumab, for squamous and non-squamous histology, respectively, represent still the backbone. The advent of the continuum maintenance strategy with pemetrexed or bevacizumab has further improved the survival outcomes of patients with adenocarcinoma histology, reaching a new plateau of about 14–16 months [5–7], while fewer options remain available for patients with squamous cell lung cancer. Unfortunately all NSCLC patients develop acquired resistance within 6–10 months of treatment, with only 20% and 8% of all diagnosed population being able to tolerate second- and third-line therapy, respectively [8].

After several years of research, new drugs have recently emerged as effective therapeutic options also in pre-treated patients who have been characterized by a particular poor prognosis and limited survival. The addition of the anti-angiogenic agents Nintedanib or Ramucirumab to docetaxel has shown a modest but significant survival benefit in patients who progressed to first-line platinum-based chemotherapy [9–14], leading to the approval of both drugs in this setting of patients. Immunotherapy, including anti-PD1 and anti-PD-L1 monoclonal antibodies (MoAbs), represents another promising strategy, which shows a significant superiority compared to standard chemotherapy in pre-treated NSCLC patients [15–17], while ongoing studies are exploring its potential application also in first-line setting.

However we are still far from their introduction into clinical practice. Therefore the current treatment options available in second-line treatment include docetaxel, pemetrexed, and the EGFR-TKI erlotinib, which is the only one approved also as third-line therapy, as highlighted by the BR.21 study, first showing a clinical benefit of erlotinib over placebo in a pre-treated and unselected NSCLC population [18]. After this trial several studies included in two recent meta-analyses compared erlotinib to standard second-line chemotherapy, such as docetaxel and pemetrexed, showing conflicting results [19–27]. Most of such studies included an unselected population with an unknown EGFR status, showing no significant differences between the two treatment arms in all patients' outcomes [28]. However also the results of the studies performed in EGFR wild-type (WT) patients are controversial [29, 30], suggesting a potential survival benefit in favor of chemotherapy [31], but highlighting the urgent need of direct comparisons between TKI and chemotherapy in this setting of NSCLC patients. We compared clinical outcomes of erlotinib and of single-agent chemotherapy in EGFR-WT or unknown NSCLC patients who progressed to first-line platinum-chemotherapy.

In EGFR wild-type NSCLC the EGFR pathway is activated, even though the excessive activity of kinase domain as in the mutant EGFR receptor lacks. This explains why EGFR driver mutations have an oncogenic effect. The modestly activated non-mutated EGFR has anyways a role in cancer promotion by the induction of cell proliferation, apoptosis inhibition, angiogenesis, cellular motility and invasiveness [32].

The aim of this study was to demonstrate the best second-line treatment option in a real-life EGFR-WT or unknown NSCLC population, trying to identify a preferred sequence of treatment, considering at least two or three lines of therapy and alternating respectively chemotherapy and TKI. The results could help to understand the role of EGFR-TKI in the sequence strategy. This information could help decision making in light of the new options for second-line treatment in EGFR-WT patients.

## RESULTS

### Patients characteristics

93 patients have met the criteria and have been selected for the first evaluation. 69 were male while 24 female and the mean age at diagnosis was 66,7 (range 46–84). 39 EGFR-WT and 54 EGFR-UK. All patients received a first-line chemotherapy and a TKI as second- or third-line treatment: 80 among them were treated with a platinum-based doublet chemotherapy, while 13 with single-agent chemotherapy. All patients received at least one line of EGFR-TKI: 67 as second-line and 26 as third-line treatment after second-line chemotherapy. Table 3 and Figure 3 summarize the main characteristics of the 93 cases analyzed. Among patients treated with TKI as second-line treatment, 13 received gefitinib and 54 erlotinib. Among patients treated with TKI as third-line treatment all received erlotinib. The following analyses were conducted comparing two defined groups of patients: patients treated in sequence with two lines of chemotherapy followed by TKI in the third (CT-CT-TKI) and patients treated with first-line chemotherapy, TKI in second and subsequent third-line chemotherapy (CT-TKI-CT). We evaluated TTP of the second-line and the third-line treatment, and OS. For the comparison of these main endpoints (TTP and OS) for the second-line treatment, we included data even from patients treated with only two lines of treatment (CT-TKI).

**Table 1:** Baseline patients' characteristics

Characteristics	*N* (%)
**Mean age at diagnosis (range)**	66,7 (46**–**84)
< 65	34 (36,6)
≥ 65	59 (63,4)
**Sex**	
M	69 (74,2)
F	24 (25,8)
**Performance status**	
0–1	86 (92,5)
2	7 (7,5)
**Smoking status**	
Yes	42 (45,2)
No	18 (19,3)
Former	33 (35,5)
**Histology**	
Squamous	24 (25,8)
Adenocarcinoma	56 (60,2)
Other	13 (14,0)
**Stage at diagnosis**	
I–IIIA	9 (9,7)
IIIB–IV	84 (90,3)
**Comorbidity**	
Yes	56 (60,2)
No	37 (39,8)
**Number of metastatic sites**	
1	32 (34,4)
2	37 (39,8)
≥ 3	24 (25,8)
**EGFR**	
Wild-type (WT)	39 (41,9)
Unknown (UK)	54 (58,1)

**Table 2:** TTP of second- and third-line treatment (TKI vs CT)

	TKI		CT		HR	95% CI	*P*
	mTTP (months)	*N*	mTTP (months)	*N*			
Second line	5	58	4	26	0,91	0,57–1,45	0,6333
Third line	2	22	3	25	1,1	0,62–1,97	0,6951

**Table 3:** Overall survival according to TKI position (3rd-line vs 2nd-line) and overall sequence (CT-CT-TKI vs CT-TKI-CT)

	Treatment	mOS (months)	N	HR	95% CI	P
TKI position	3rd-line TKI	26	22	0,68	0,42 – 1,09	0,11
	2nd-line TKI	15	51			
Overall sequence	CT-CT-TKI	26	21	0,70	0,39 – 1,24	0,21
	CT-TKI-CT	18	26			

### TTP of second- and third-line treatment (TKI vs CT)

**Figure 1:**
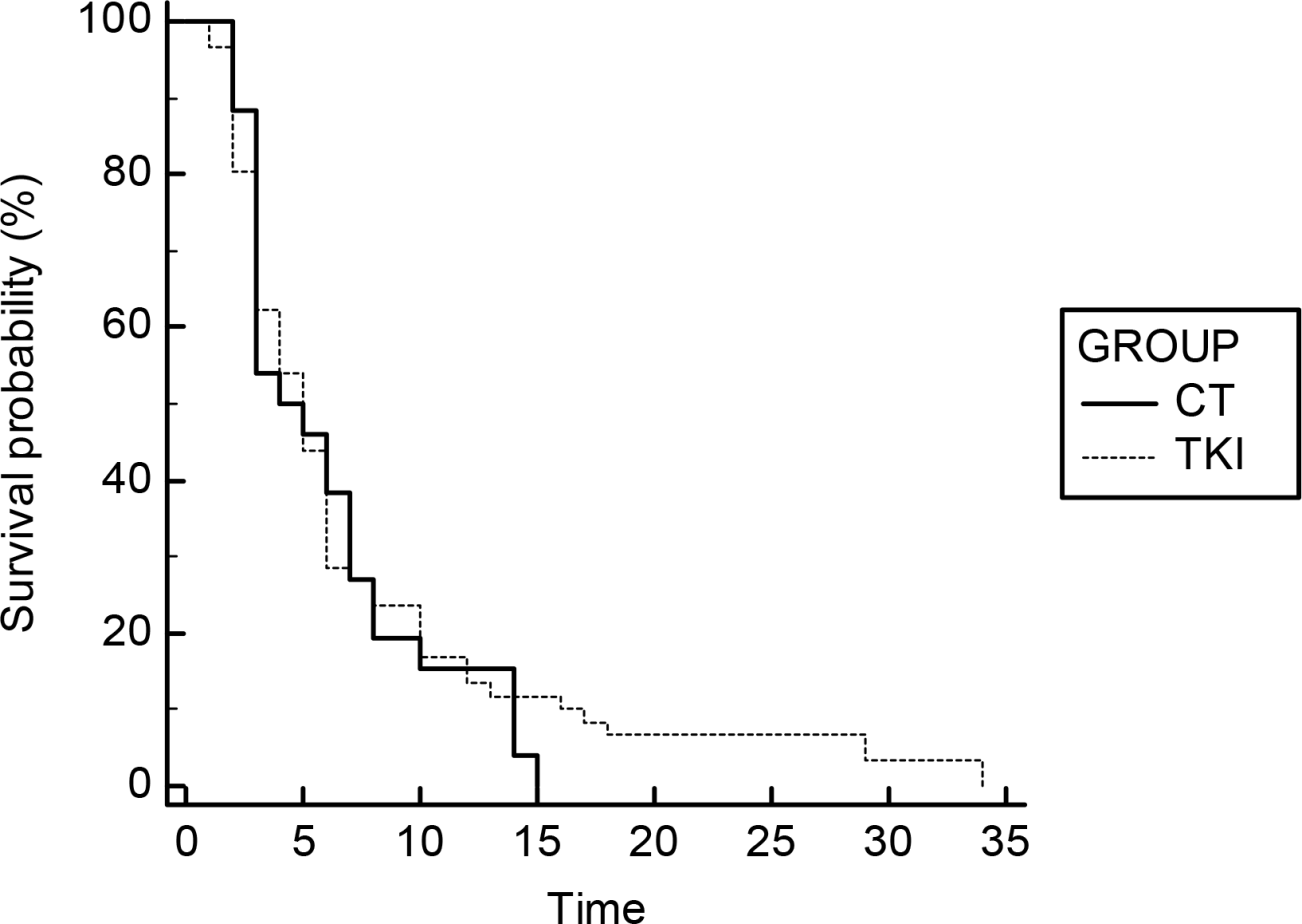
Survival curves of TTP for second-line treatment (TKI vs CT)

**Figure 2:**
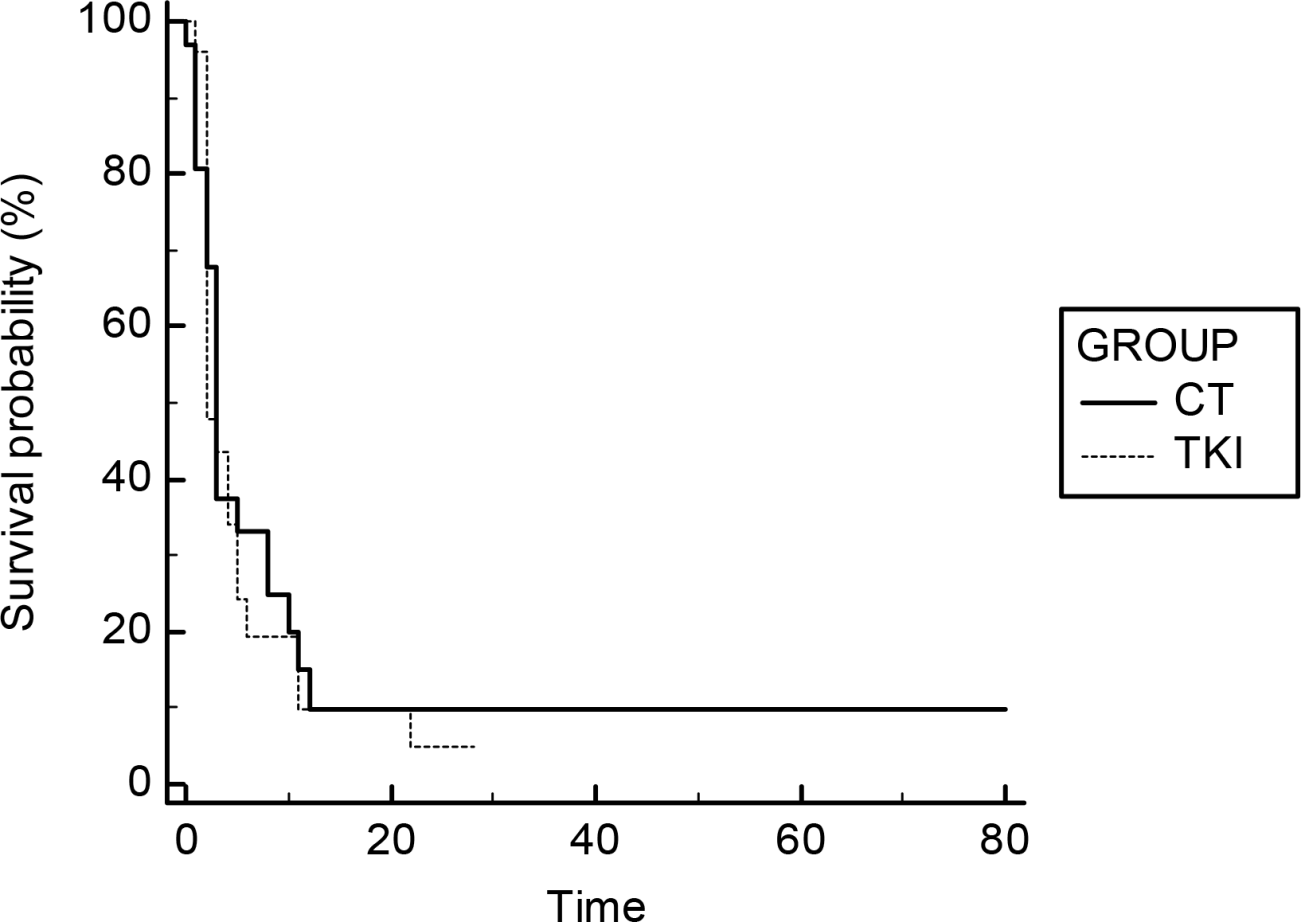
Survival curves of TTP for third-line treatment (TKI vs CT)

No statistically significant difference was found, in terms of TTP, for EGFR-WT or EGFR-UK patients treated with TKI or chemotherapy as second-line treatment: mTTP 5 vs 4 months; HR: 0,91 (TKI vs CT) 95% CI: 0,57–1,45; *p* = 0,6333 (Figure 1). Similarly, the analysis of the TTP of the third-line treatment confirms the substantial overlap of the two strategies (TKI and CT): mTTP 2 vs 3 months; HR: 1.1; (TKI vs CT); 95% CI: 0.62 to 1,97; *p* = 0,6951. Even in this graph, the curves representing the two treatments under comparison are almost identical, although a fewer number of patients continue to benefit, to date, by such therapies (Figure 2). The Table 2 summarizes these data.

### Overall survival

**Figure 3:**
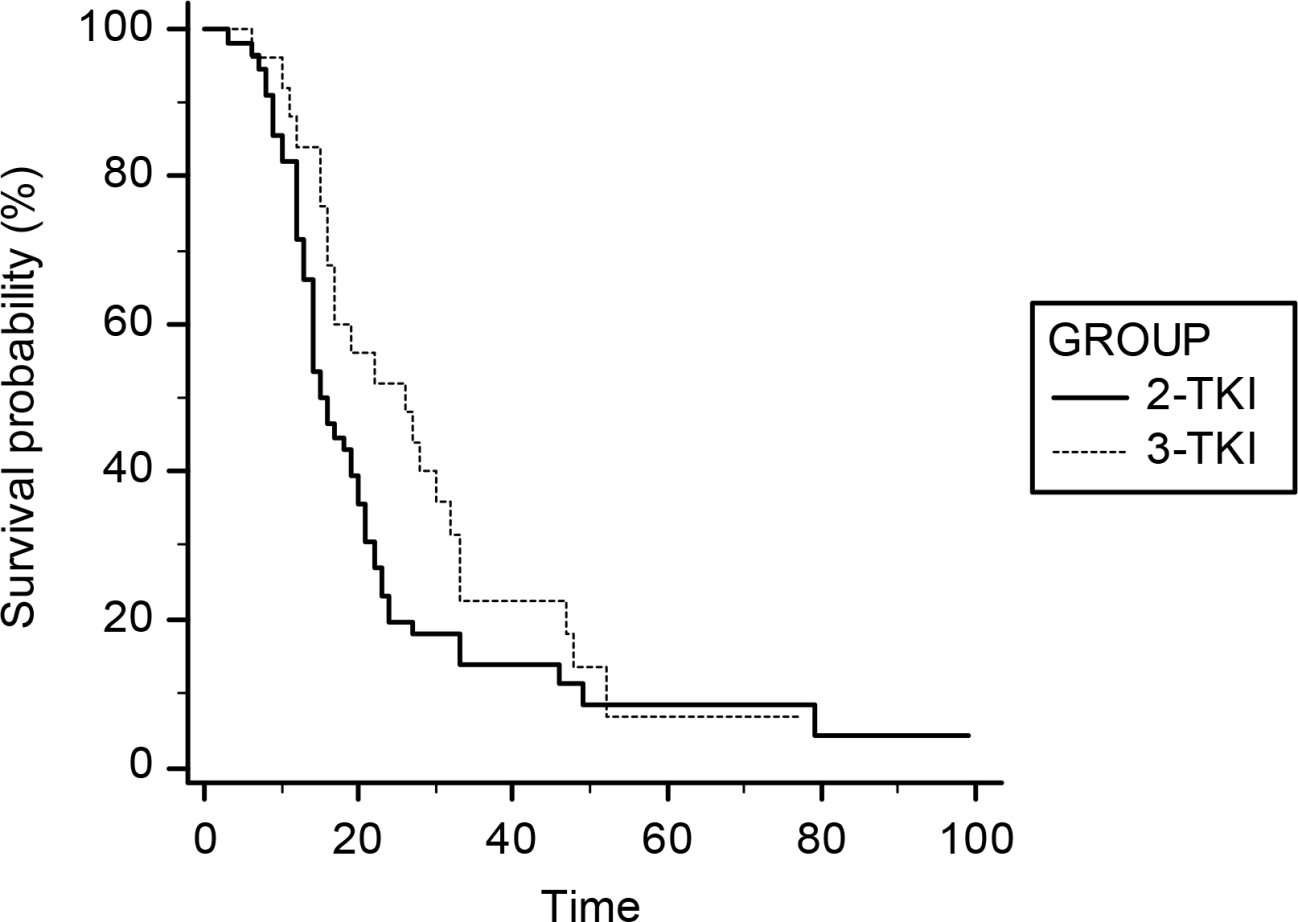
Survival curves of OS for the comparison 3rd-line vs 2nd-line TKI

**Figure 4:**
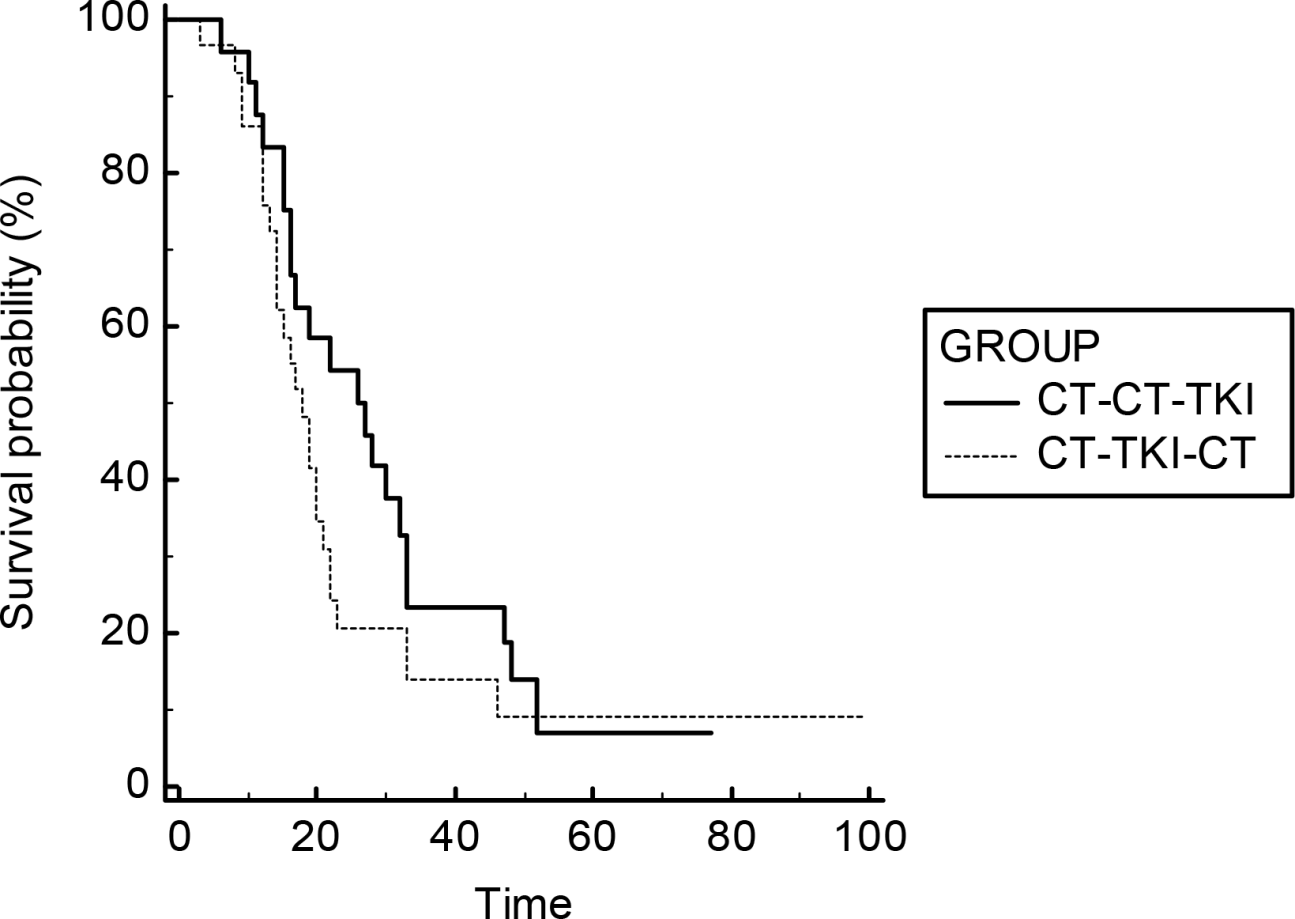
Survival curves of OS for the comparison CT-CT-TKI vs CT-TKI-CT

For OS analysis we compared EGFR-WT and EGFR-UK patients treated with TKI as second- (CT-TKI) or third-line therapy (CT-CT-TKI) respectively. The results showed a trend toward a benefit for patients in the third-line TKI group: mOS 26 vs 15 months (third-line TKI vs second-line TKI), HR: 0,68, 95% CI: 0,42–1,09; *p* = 0,11. No statistical significance have been found (Figure 3). Among patients treated with TKI as second-line treatment, a consistent number of them (*N* = 40) were treated only for two lines of therapy, showing lower survival (Table 3). However in the survival comparison between patients who have been treated with at least three lines of therapy (CT-CT-TKI vs CT-CT-TKI), a statistically non-significant trend has been also confirmed in favor of the TKI in the third-line treatment (mOS 26 vs 18 months; HR: 0,7; *p* = 0,21) (Figure 4 and Table 3).

## DISCUSSION

This retrospective analysis has been carried out in order to find confirmation of the rationale of EGFR-TKI use for EGFR-WT and EGFR-UK NSCLC patients in every day clinical practice. Although the best outcomes are obtained from the use of TKIs as upfront treatment in EGFR mutated patients, all the international guidelines agree to recommend erlotinib as potential treatment option even for EGFR-WT patients who progressed to first-line platinum-chemotherapy [33]. Indeed a modest activation of the EGFR signaling pathway has been demonstrated also in EGFR-WT NSCLC, promoting cancer cells proliferation, motility and invasiveness, stimulating tumor angiogenesis and inhibiting apoptosis processes [32, 34]. Additionally several phase II studies have shown encouraging activity and survival outcomes of erlotinib as second/third-line treatment in EGFR-WT NSCLC patients [35–37], whereas the randomized phase III BR.21 study demonstrated a significant benefit of erlotinib over placebo in a pre-treated and unselected NSCLC population [18], leading to its approval in such setting in 2004. A real-word cost-effectiveness analysis was conducted in NSCLC patients who received erlotinib after prior chemotherapy-regimens, suggesting it as a cost-effective treatment in this setting of patients [38]. However it has not been clarified yet the best allocation of EGFR-TKI (second- or third-line setting) in the treatment strategy of such patients.

This study was intended for the assessment of the efficacy of EGFR-TKIs (erlotinib or gefitinib) compared to standard single-agent chemotherapy (docetaxel or pemetrexed) as second- or third-line treatment in EGFR-WT/UK NSCLC population. The aim was the finding of support to identify a preferred sequence of treatment for these patients. Overall the baseline patients' characteristics in the present study were similar to those reported in other prospective randomized studies, including the high percentage of both adenocarcinoma histology and good PS [18, 25, 39]. The results of our analyses showed the substantial equality in terms of TTP of the two treatment strategies (TKI vs CT) both in second- and in third-line setting. Such data are opposite to the results from the most recent published randomized trials, including TAILOR, CTONG0806 and DELTA studies [25–27]. All of these ones show a significant superior PFS in favor of chemotherapy over TKI in EGFR-WT NSCLC patients who progressed to first-line treatment. Such discordance could be explained by the high percentage (58%) of patients with an unknown EGFR mutational status included in our study. Another possible explanation may entail the lower sensitivity of the techniques used for the assessment of EGFR mutational status in our study, compared to the advanced platforms with higher sensitivity used in more recent trials (i.e. Sanger Sequencing vs Next-Generation Sequencing, respectively). As regards the OS analysis, our results suggest that treatment with EGFR-TKI is not inferior to the standard single-agent chemotherapy. Trends toward better OS were observed with third-line EGFR-TKI in both the comparisons (CT-TKI vs CT-CT-TKI and CT-TKI-CT vs CT-CT-TKI), although such differences were not statistically significant. According to other recently published randomized trials [25, 26], such evidences suggest a potential superiority of second-line chemotherapy over EGFR-TKI in NSCLC patients with EGFR-WT/UK, which failed to reach a statistical significance because of the effect of the treatment cross-over on the outcomes. Furthermore we can affirm that the potential OS benefit in favor of second-line CT is not induced by the number of treatment lines, but it's likely related to the right sequence of drugs. Anyway the significance of results in a retrospective analysis remains uncertain. Indeed as a retrospective study, our work has several limitations, primarily the small number of subjects included as well as the high percentage of patients with an unknown EGFR mutation status, which of course reduce the reliability of our data. Analyzing our results we can conclude that, as recommended by all the international guidelines, both chemotherapy and EGFR-TKI are equal treatment options for NSCLC patients who progressed to first-line platinum-based chemotherapy. However for those patients with good PS who are fit to receive it, chemotherapy could represent the preferred second-line option, reserving EGFR-TKI as third-line therapy. Similarly EGFR-TKI, thanks to its peculiar tolerability profile can be considered as a valid alternative to chemotherapy for those patients with a poor PS or other comorbidities, who are unable to tolerate single-agent chemotherapy but are eligible for a second-line treatment. Moreover the LUX-Lung 8 trial compared afatinib with erlotinib, as second-line treatment for patients with advanced squamous NSCLC. PFS and OS were improved by afatinib. So this irreversible EGFR-TKI should be considered as an alternative option in this setting [40].

Recently several studies investigated the potential role of a “proteomic scoring” by mass spectrometry analysis of serum to identify EGFR-WT patients most likely to benefit from EGFR-TKI [41–43]. Recently a phase III randomized study has shown that patients with a “poor proteomic score” had inferior OS with EGFR-TKI compared to chemotherapy [44]. Other studies evaluated germline genetic polymorphisms as predictors of response to EGFR-TKIs, showing that genetic polymorphisms of EGFR and downstream PI3K/AKT signaling pathways may be surrogate biomarkers of EGFR-TKI activity and toxicity in NSCLC patients [45–48]. Such data suggest that there is a high inter-patient variability likely related to their pharmacogenetic heterogeneity. However such findings have not been translated into clinical practice yet. Therefore clinical criteria, including patient's age, PS, comorbidities and toxicities, remain still crucial in the choice of the best second-line treatment, which should be individualized according to every patient's characteristics and preferences.

## CONCLUSIONS

This study, far to draw any definitive conclusions, is a report of real-life clinical practice, which aims to stimulate the current open debate on the best treatment algorithm in EGFR-WT NSCLC patients, highlighting the urgent need for further studies on this topic. EGFR-TKI should be considered as a treatment option after progression upon progression to first-line treatment. Probably the advent of new drugs recently approved in second-line setting will lead to new treatment algorithms, but it is still far from clinical practice. In this framework this study could support the use of EGFR-TKI as third-line treatment option after new second-line treatment regimens.

## MATERIALS AND METHODS

### Objectives of the study

Primary objective of the study has been to determine what is the most appropriate treatment between TKI and chemotherapy in EGFR-WT (ascertained non-mutated EGFR status) or EGFR-UK (unknown EGFR mutational status) advanced NSCLC patients, after progression from a first-line chemotherapy. The two second-line options would be compared on the basis of efficacy data: time to progression (TTP) and overall survival (OS). The same comparison would be also carried out for the third-line treatment.

Secondary objective of the study was to evaluate the influence of activity and effectiveness of the first-line chemotherapy, and the activity and effectiveness of TKI as second-line treatment. To address this objective we would analyze the association between the first-line chemotherapy responders and responders to the second-line treatment with TKI. As “responders” we mean those patients that have shown complete (CR), partial response (PR) or stable disease (SD) lasting six months or more. Conversely “non-responders” are those patients who have shown progressive disease (PD) or SD ≤ 6 months. In addition, we would evaluate the influence of the TTP to first-line chemotherapy on the TTP to second-line treatment with TKI.

### Study design

This study was carried out as a retrospective analysis by data collected from medical records of patients with histologically confirmed diagnosis of locally advanced or metastatic (Stage IIIB–IV) NSCLC and treated for at least two lines of therapy and at least one line of EGFR-TKI. We evaluated patients treated from 2005 to present in various cancer centers in Italy. Patients with EGFR mutations were excluded and we selected for the analysis only EGFR-WT and EGFR-UK patients evaluating the second- and third-line treatment, after first-line chemotherapy.

### Eligibility criteria

The patients were included for the analysis if they met the following eligibility criteria.

Inclusion criteria:

- Histologically confirmed diagnosis of stage IIIB– IV NSCLC;
- History of at least two lines of treatment for the advanced disease, with at least an instrumental re-evaluation of the disease after at least two months for each line of treatment;
- History of at least one line of treatment with a TKI.

Exclusion criteria:

- Early stage NSCLC;
- EGFR mutated status;
- Treatment with TKI after the third-line treatment;
- Interruption of any of the treatments before two months from the beginning.

### Data collection

Data would be collected from paper or electronic databases after the obtainment of written informed consent to cancer treatment and processing of personal data. The following characteristics were evaluated for every single patient.

Patient's data: 1) sex; 2) age at diagnosis; 3) smoking status; 4) Eastern Cooperative Oncology Group Performance Status (ECOG PS) at diagnosis; 5) Comorbidities. Disease's data: 1) histotype; 2) stage at diagnosis (IIIB or IV); 3) sites of metastases; 4) EGFR mutational status (EGFR-WT or EGFR-UK); 5) type of EGFR mutation. Treatments and outcomes: 1) type of treatment (schedules and drugs); 2) best objective responses (complete response (CR), partial response (PR), stable disease (SD), progressive disease (PD), according to RECIST criteria); time-to-progression (TTP), meant as the time in months from the beginning of any treatment and progression; overall survival (OS) meant as the time in months from the beginning of the treatments and death or the last visit.

### Statistical analysis

The analyses were performed by MedCalc version 14.12.0. For the comparison of the effectiveness of CT and TKI at second- and third-line for TTP and OS, we performed the Kaplan-Meyer test with use of the log-rank test to calculate median TTP (mTTP) and median OS (mOS). For all statistical tests, a *p* < 0.05 has been considered as statistically significant.
